# MiR-10b decreases sensitivity of glioblastoma cells to radiation by targeting AKT

**DOI:** 10.1186/s40709-016-0051-x

**Published:** 2016-06-24

**Authors:** Limin Zhen, Jian Li, Mingran Zhang, Kun Yang

**Affiliations:** Department of Neurosurgery, Taian Central Hospital, No. 29, Longtan Road, Taian City, Shandong Province China

**Keywords:** MiR-10b, Glioblastomas, Apoptosis, AKT

## Abstract

**Background:**

Glioblastomas are the most aggressive brain tumors with extremely poor prognosis despite advances in treatment techniques. MiR-10b is highly expressed in glioblastoma and regulates cell proliferation, migration and invasion. Here, we examined the role of MiR-10b on radiotherapy of glioblastomas.

**Methods:**

MiR-10b mimic or anti-MiR-10b inhibitor was transfected in glioblastoma cells. WST-1 assay was used to examine the effect of MiR-10b on proliferation of transfected glioblastoma cells after radiation treatment. Apoptosis was examined by caspase 3/7 activity and TUNEL assay. The western blot was used to evaluate protein expression.

**Results:**

Altered expression of MiR-10b changed the radiation-induced inhibitory effect on proliferation of glioblastoma cells with dose-dependent manner. MiR-10b decreased radiation-induced apoptosis in glioblastoma cells by activation of caspase 3/7 and inhibition Bcl-2 expression. MiR-10b enhances migration and invasion of glioblastoma cells in presence of radiation. In addition, MiR-10b decreased the sensitivity of glioblastoma cells to radiotherapy by activation of p-AKT expression.

**Conclusions:**

MiR-10b might be a potential biomarker to predict radiotherapy response and prognosis in glioblastomas.

## Background

Glioblastomas remains the most common malignant primary brain tumor in adults [1]. These tumors account for 17 % of all intracranial tumors and 55 % of astrocytic tumors with an estimated 12,120 new cases predicted in 2016 [2]. Glioblastomas are highly aggressive glioma with median survival rate of less than 15 months and 5-year survival rate of less than 4 % and are characterized by rapid growth, invasion and angiogenesis [3]. Glioblastomas arise from astrocytes, and contain a mix of cell types histologically, which makes the treatment difficult [4]. The most common symptoms in patients with glioblastomas are caused by increased pressure in the brain, such as headache, nausea and vomiting [5]. Although progress in treatment techniques, including surgery, radiotherapy and chemotherapy, the median survival for patients with glioblastomas has only marginally changed [6]. Therefore, it is critical to find out new molecular targets and approaches to treat this aggressive disease. Radiotherapy is the standard adjuvant treatment for glioblastoma to kill remaining tumor cells after surgical removal [7]. It is also used to treat unresectable tumors combined with chemotherapy. Recent studies have shown some agents may be used as radiosensitizers or radiation modulators and enhance the efficacy of radiotherapy in tumor treatment [8].

MicroRNA expression profiling studies have revealed that numerous microRNAs (MiRNAs) are associated with glioblastoma tumorigenesis [9]. MiRNAs are short, non-coding RNA molecules and play important role in the regulation of gene expression by directly binding to target sites in the 3′-untranslated region of the targeted mRNA. MiRNAs can function as either tumor suppressors by targeting oncogenes or oncogenes by targeting tumor suppressors [10]. Recently, growing evidence has shown that MiR-10b is highly expressed in glioblastoma and could be a potential therapeutic target [11, 12]. MiR-10b can promote cell cycle progression, migration and invasion of glioma cells [12, 13]. Further studies have demonstrated that MiR-10b regulates different gene expression and signaling pathways in heterogeneous cellular environments [14].

Here, we examined the roles of MiR-10b in radiotherapy of glioblastoma. We found that overexpression of miR-10b decreased the radiation-induced inhibitory effect on proliferation of glioblastoma cells, while down regulation of MiR-10b increased the radiation-induced inhibitory effect on proliferation of glioblastoma cells. Meanwhile, overexpression of MiR-10b inhibited radiation-induced apoptosis, promoted migration and invasion on glioblastoma cells by activation of AKT signaling. Our findings indicate that MiR-10b could be a potential biomarker to predict the radiotherapy response and prognosis in glioblastomas.

## Results

### MiR-10b alters the radiation-induced inhibitory effect on proliferation of glioblastoma cells

**Fig. 1:**
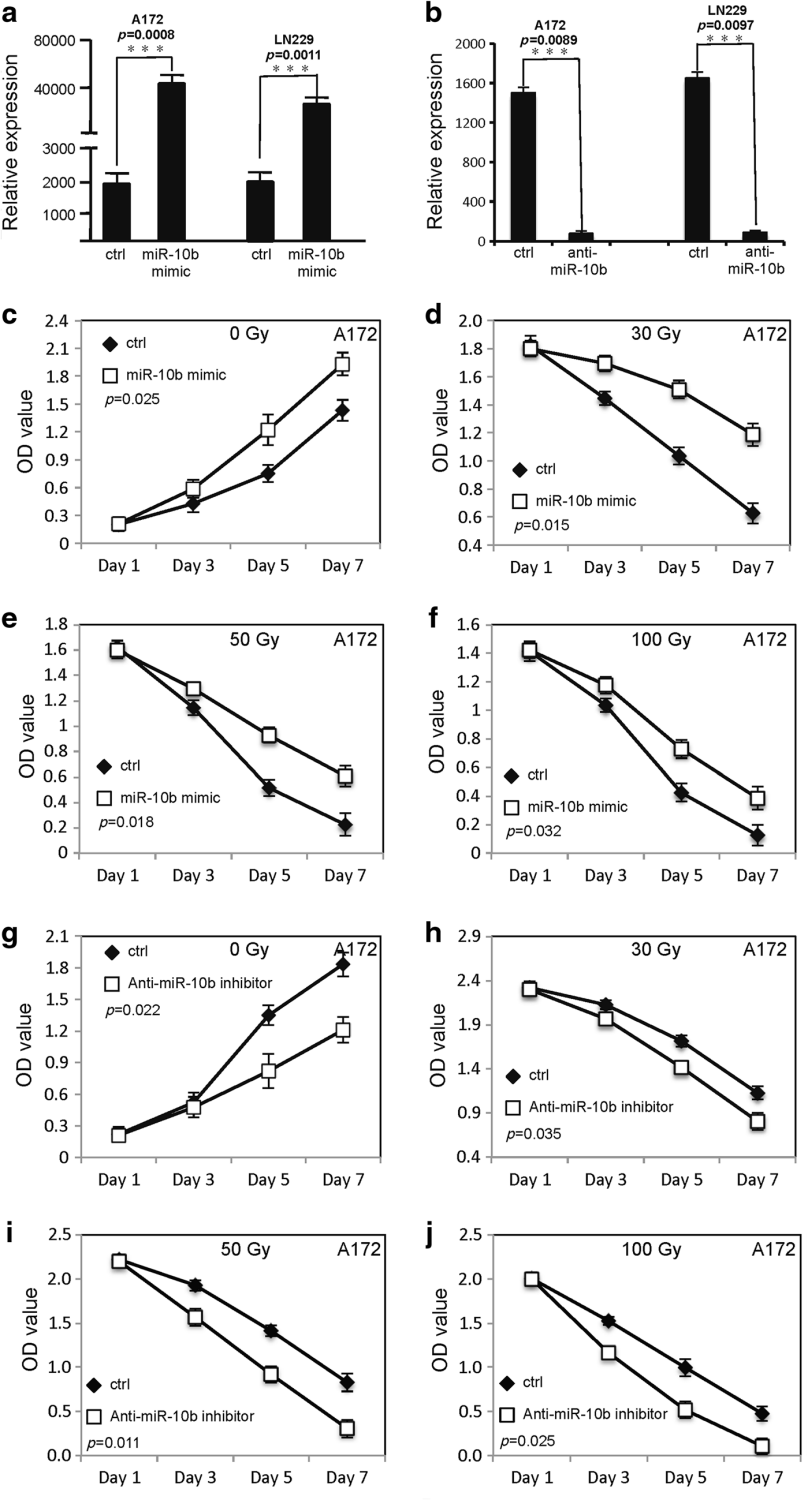
MiR-10b promotes the survival of glioblastoma cells following irradiation. **a** The MiR-10b expression level in glioblastoma cells transfected with MiR-10b mimic. **b** The MiR-10b expression level in glioblastoma cells transfected with anti-MiR-10b inhibitor. **c**–**f** The proliferation of A172 cells transfected with MiR-10b mimic or random sequence MiRNA mimic molecule after different doses of irradiation treatment. **g**–**j** The proliferation of A172 cells transfected with anti-MiR-10b inhibitor or random sequence MiRNA mimic molecule after different doses of irradiation treatment

**Fig. 2:**
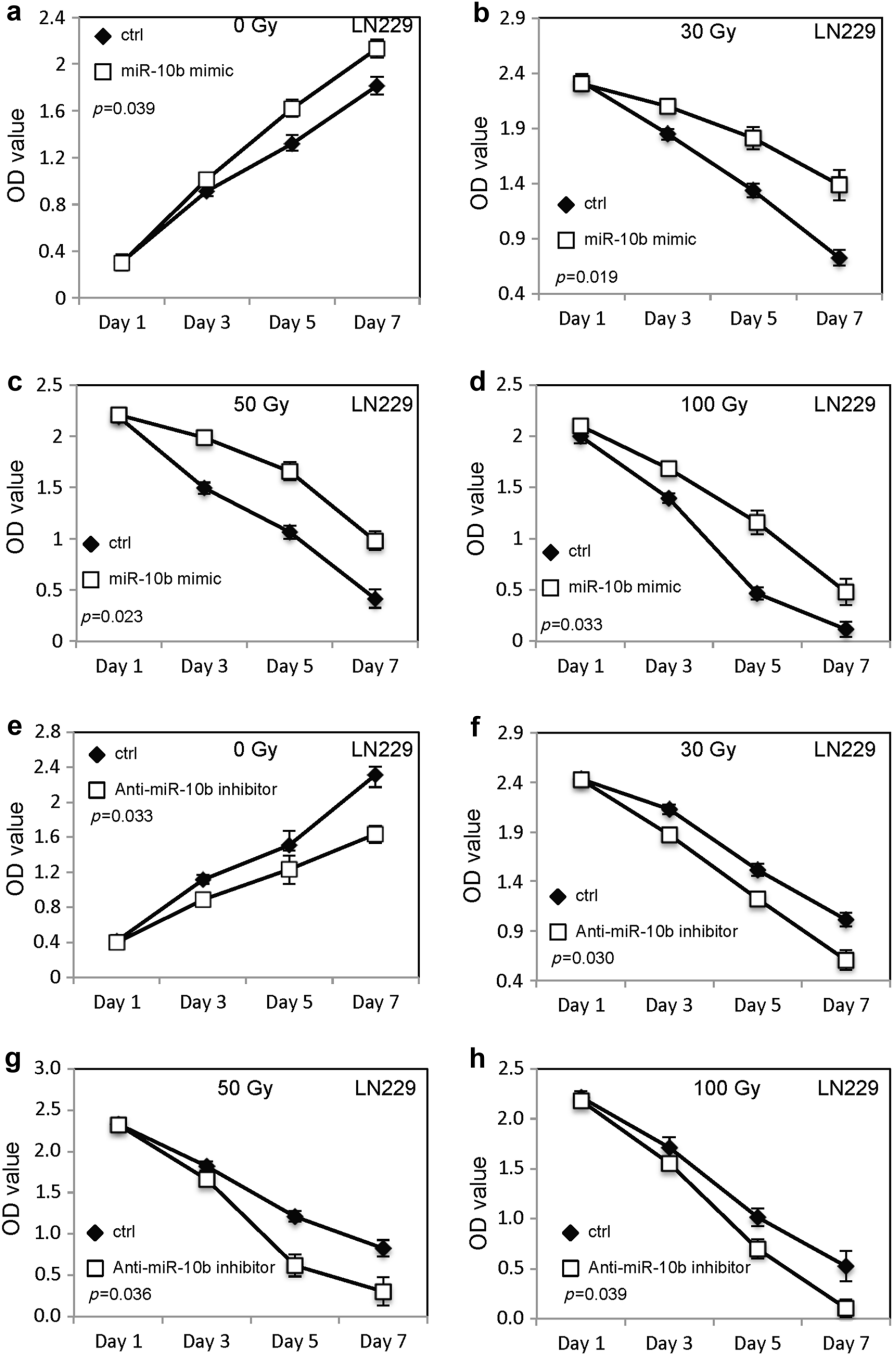
MiR-10b promotes the survival of glioblastoma cells following irradiation. **a**–**d** The proliferation of LN229 cells transfected with MiR-10b mimic or random sequence MiRNA mimic molecule after different doses of irradiation treatment. **e**–**h** The proliferation of LN229 cells transfected with anti-MiR-10b inhibitor or random sequence MiRNA mimic molecule after different doses of irradiation treatment

To examine the role of MiR-10b in the proliferation of glioblastoma cells, MiR-10b mimic or anti-MiR-10b inhibitor, and a random sequence MiRNA mimic molecule (used as a negative control) were separately transfected into glioblastoma cells followed by different doses of ionizing radiation. WST-1 assay was used to evaluate the proliferation of glioblastoma cells. As shown in Fig. 1a, b, the MiR-10b expression level was shown in transfected glioblastoma cells by qRT-PCR. Overexpression of MiR-10b promotes proliferation of both A172 and LN229 cells in absence of ionizing radiation (Figs. 1c, 2a). When A172 and LN229 cells (transfected with MiR-10b mimic or a random sequence MiRNA mimic molecule) were treated with different doses of ionizing radiation (30, 50 and 100 Gy), MiR-10b decreased the radiation-induced inhibitory effect on the proliferation of A172 and LN229 cells at dose dependent manner (Figs. 1d–f, 2b–d). In contrast, down expression of MiR-10b decreased proliferation of both A172 and LN229 cells in absence of ionizing radiation (Figs. 1g, 2e). When A172 and LN229 cells, transfected with anti-MiR-10b inhibitor or a random sequence MiRNA mimic molecule, were treated with different doses of ionizing radiation (30, 50 and 100 Gy), MiR-10b enhanced the radiation-induced inhibitory effect on the proliferation of A172 and LN229 cells at dose dependent manner (Figs. 1h–j, 2f–h).

### MiR-10b inhibits radiation-induced apoptosis in glioblastoma cells

**Fig. 3:**
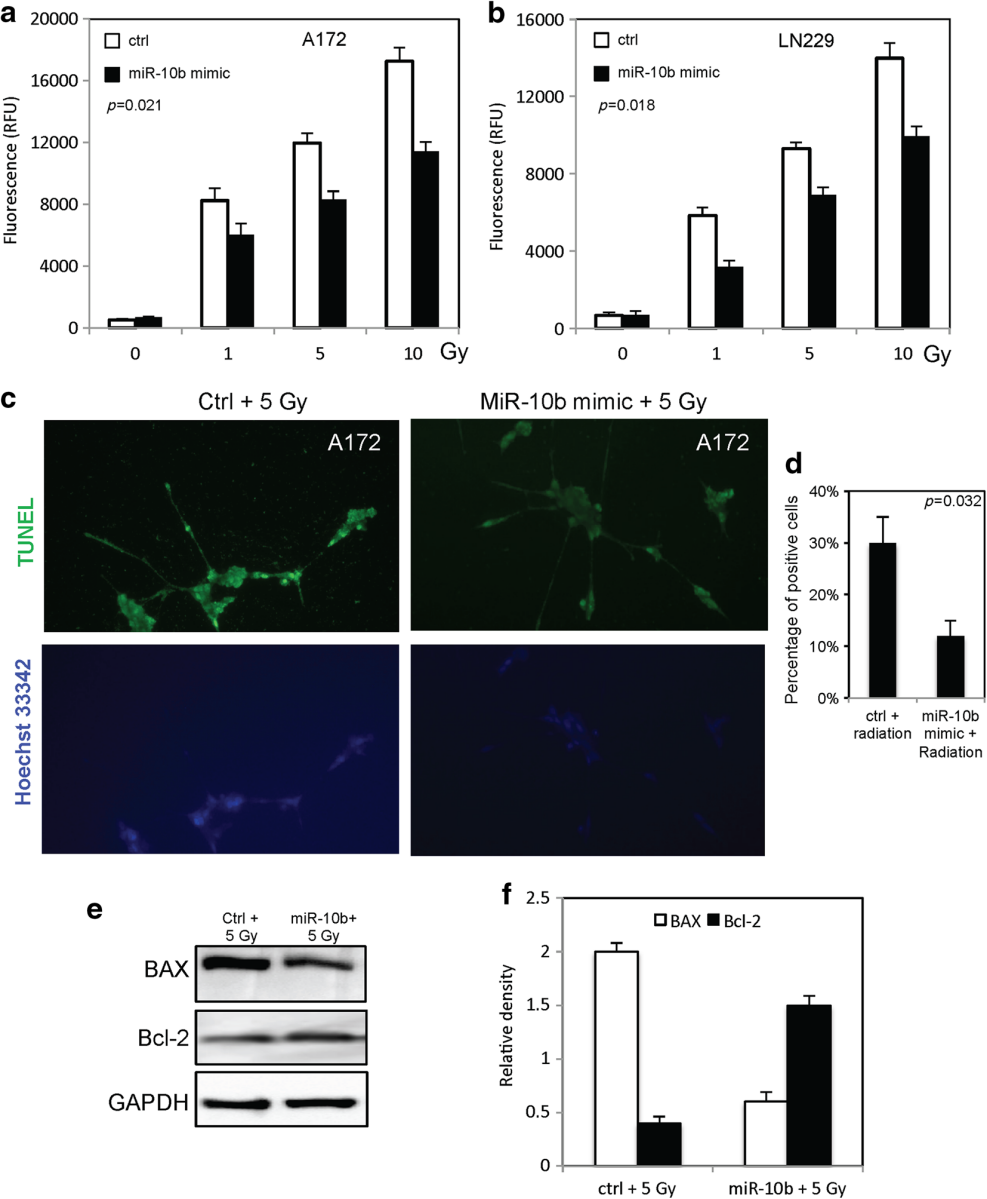
MiR-10b inhibits radiation-induced apoptosis in glioblastoma cancer cells. **a**, **b** The caspase-3/7 activity in A172 cells and LN229 cells either transfected with MiR-10b mimic or random sequence MiRNA mimic molecule. **c**, **d** The TUNEL positive A172 cells either transfected with MiR-10b mimic or random sequence MiRNA mimic molecule at 5 Gy of radiation. **e**, **f** The levels of apoptotic proteins in A172 cells either transfected with MiR-10b mimic or random sequence MiRNA mimic molecule at 5 Gy of radiation

We further examined the role of MiR-10b on radiation-induced apoptosis in glioblastoma cells by checking caspase 3/7 activity, TUNEL and apoptotic related protein. MiR-10b inhibited the caspase 3/7 activity in glioblastoma cells at dose dependent manner (Fig. 3a, b). The percentage of TUNEL positive cells significantly decreased after overexpression of MiR-10b in A172 cells (Fig. 3c, d). MiR-10b changed the expression of Bax and Bcl-2 in A172 cells (Fig. 3e, f).

### MiR-10b enhances migration and invasion in glioblastoma cells

**Fig. 4:**
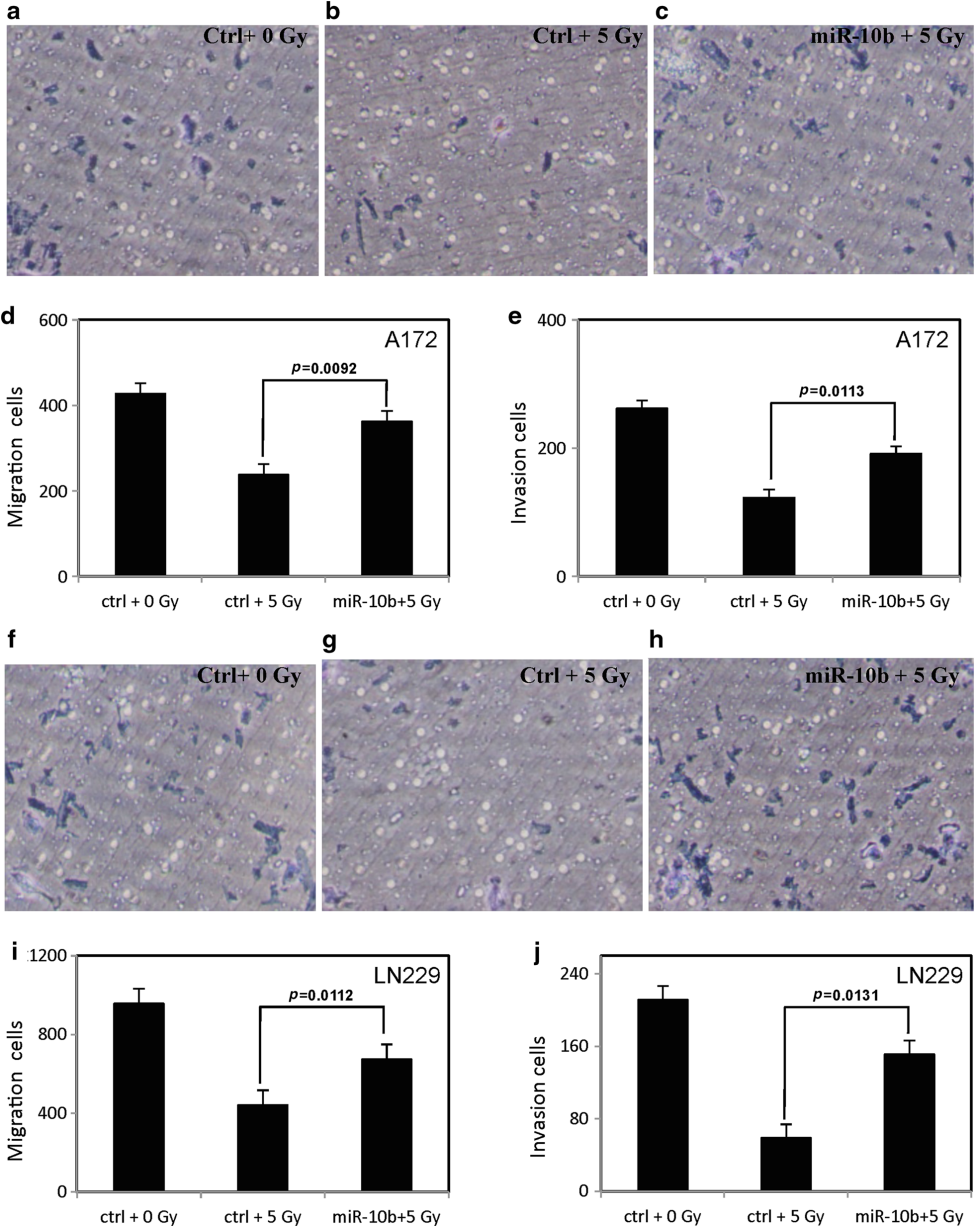
MiR-10b increases the migration and invasion of glioblastoma cells. The invasion of A172 cells (**a**–**c**) and LN229 cells (**f**–**h**) transfected with the MiR-10b mimic or random sequence MiRNA mimic molecule after 5 Gy of radiation. The *graph* indicates the migration of A172 cells (**d**) and LN229 cells (**i**) transfected with the MiR-10b mimic or random sequence MiRNA mimic molecule after 5 Gy of radiation. The migration of negative control cells without radiation treatment was used as a quality control. The *graph* indicates the invasion of A172 cells (**e**) and LN229 cells (**j**) transfected with the MiR-10b mimic or random sequence MiRNA mimic molecule after 5 Gy of radiation. The invasion of negative control cells without radiation treatment was used as a quality control

To examine the role of MiR-10b on cell aggression after ionizing radiation, the Transwell migration and Matrigel invasion assays were performed to evaluate migration and invasion of glioblastoma cells. MiR-10b enhanced the migration and invasion of A172 cells (Fig. 4a–e) and LN229 cells (Fig. 4f–j) as compared to the negative control cells at 5 Gy of radiation. The migration and invasion of negative control cells without radiation treatment was used as a quality control.

### MiR-10b increases p-AKT expression

**Fig. 5:**
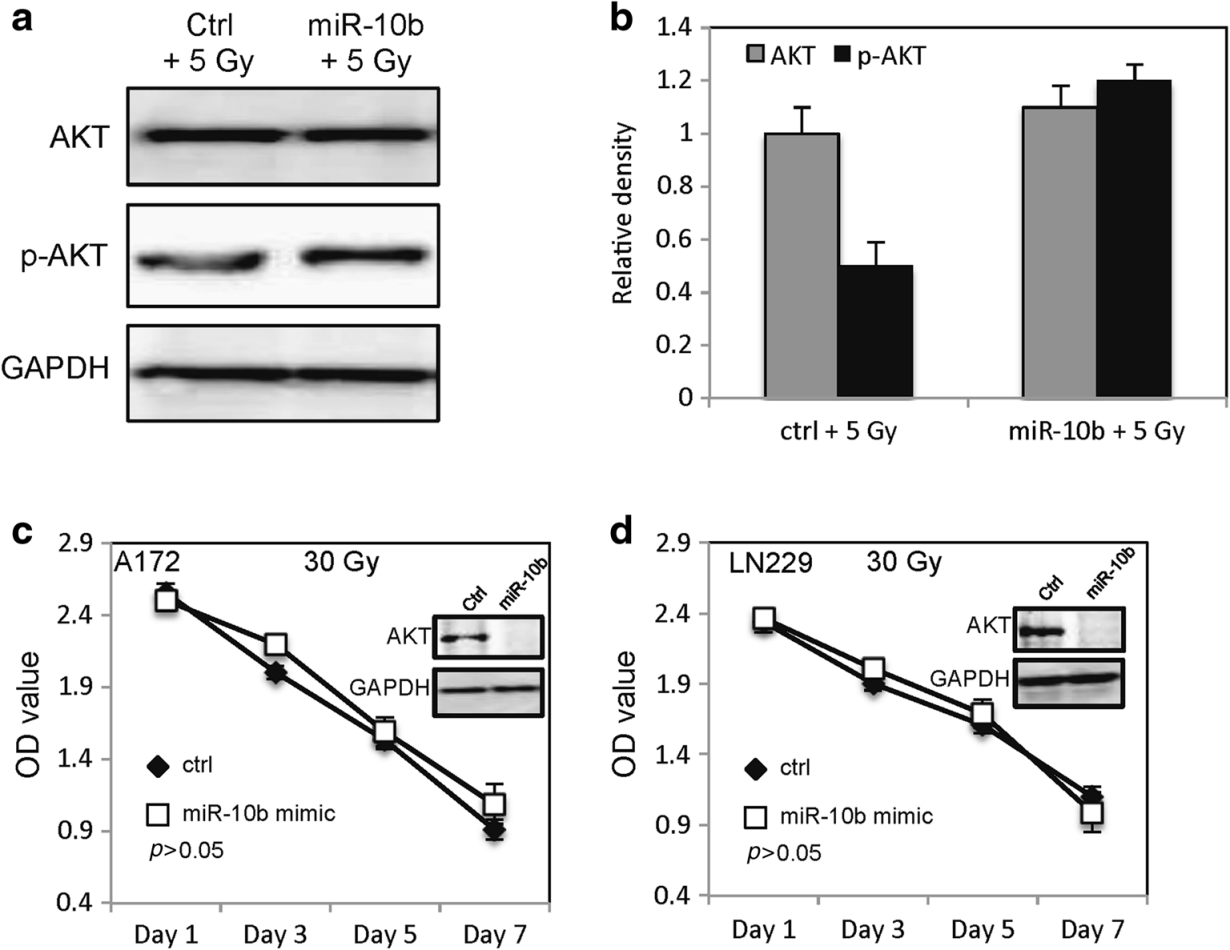
MiR-10b activates AKT in A172 cells. **a** Expression of AKT and p-AKT in A172 cells transfected with MiR-10b mimic after treated with irradiation. **b** The *graph* indicates the AKT and p-AKT relative expression level. The proliferation of A172 cells (**c**) and LN229 cells (**d**) transfected with MiR-10b mimic or random sequence MiRNA mimic molecule at 30 Gy of radiation after knockdown of AKT

We further examined the expression of p-AKT in A172 cells by western blot. As shown in Fig. 5a, b, there is no change in the total AKT expression level after overexpression of MiR-10b at 5 Gy of radiation. However, MiR-10b significantly increased p-AKT expression at 5 Gy of radiation. To further examine the role of AKT in MiR-10b mediated radioresistance of glioblastoma cells, SignalSilence AKT siRNA (catalogue number: 6211, Cell Signaling Technology, USA) was used to inhibit the expression of AKT in glioblastoma cells, then the proliferation of glioblastoma cells was examined by WST-1 assay after 30 Gy of radiation. Interestingly, when the AKT was knockdown, there is no change in the proliferation of A172 and LN229 cells transfected with MiR-10b mimic or a random sequence MiRNA mimic molecule at 30 Gy of radiation (Fig. 5c, d).

## Discussion

Radiotherapy plays central role in the treatment of brain tumors [15]. In the past decades, the standard treatment for patients with glioblastomas has been surgery with adjuvant radiotherapy. Despite the advance in efficacy of radiotherapy, various new chemotherapy agents and targeted therapies, the survival of patients with glioblastomas remains poor [16]. Meanwhile, the radiotherapy resistance is one of the reasons for the failure of treatment in glioblastoma. Although the underlying mechanism of radiotherapy resistance is poorly understood, recent studies have shown numerous biological processes alter the efficacy of radiation, such as factors and molecules in cell cycle, inactivation of tumor suppressor genes and activation of oncogenes [17, 18]. Growing evidence has demonstrated that the presence of glioma initiating cells (GICs) is associated with radiotherapy resistance [19–21]. The GICs process a faster rate of double-strand break repair caused by ionizing radiation by activation of the DNA damage response, a biological process regulated at the post-translational level. MiRNAs are also involved in DNA damage response by different mechanisms. For example, DNA damage activates PI3 K-like kinases, which triggers expression of some MiRNA [22]. MiR-10b has been reported to be highly expressed in many cancers. Altered expression of MiR-10b affects proliferation, migration and invasion of numerous cancer cells [23–25]. Preis et al. reported that increased miR-10b expression was associated with resistance to chemotherapy and radiation in pancreatic ductal adenocarcinoma cells [26]. In our study, we found that MiR-10b overexpression decreased the radiation-induced inhibitory effect in glioblastoma cells. These results extent the critical role of MiR-10b in glioblastoma cells.

Another reason that makes glioblastomas untreatable is due to their highly invasive activity. These tumors can infiltrate adjacent healthy brain and make it less possible to be fully resected during surgery. It has been demonstrated that some growth factors mediate invasion of glioblastoma, such as transforming Growth Factor-beta (TGFβ). Liu et al. found that TGFβ might induce MiR-10b expression and involved in the TGFβ-mediated migration of brain tumor cells by targeting PTEN [27]. Here, our study showed that MiR-10b enhanced the migration and invasion of glioblastoma cells after ionizing radiation. These findings suggest that MiR-10b has essential functions in glioblastoma progression.

AKT regulates multiple biological processes such as apoptosis, cell proliferation and cell growth. AKT is a serine-threonine protein kinase, and phosphorylation at S473 [28] and T308 [29] activates AKT, which mediates the downstream responses including cell proliferation, apoptosis and metabolism [30]. Liu et al. [27] found that MiR-10b could suppress PTEN expression. Gabriely et al. [12] found that MiR-10b regulated apoptosis of glioma cells by targeting BCL2 signaling. Our results showed that MiR-10b increased p-AKT expression in both presence and absence of ionizing radiation. These results indicated that AKT signaling pathway might be involved in the regulation of MiR-10b on the proliferation and apoptosis of glioblastoma cells after ionizing radiation treatment. Further studies are needed to elucidate the underlying molecular mechanism.

## Conclusion

MiR-10b plays critical role in the regulation of tumorigenesis and malignant progression of glioblastoma. Our results indicate that MiR-10b might be a potential biomarker to predict the radiotherapy response and prognosis in glioblastomas.

## Methods

### Cell lines

A172 and LN229, human glioblastoma cell lines were purchased from American Type Culture Collection (ATCC, USA). The tumor cells were cultured in Dubelcco’s modified Eagle’s medium (DMEM) (Invitrogen, USA) supplemented with 100 µg ml^−1^ streptomycin, 100 U ml^−1^ penicillin and 10 % fetal bovine serum (Invitrogen, USA). The cells were grown at 37 °C in a humidified incubator with 5 % CO_2_. All cells used in our experiments were at 70–80 % confluence.

### Cell transfection and MiRNA quantification

MiR-10b mimics or anti-miR-10b inhibitor (Catalogue numbers 4464066 and 4464084, respectively, Invitrogen, USA) was transfected to glioblastoma cells using Lipofectamine 2000 (Invitrogen, USA) following the manufacturer’s instructions. A random sequence MiRNA mimic molecule was used as a negative control (mirVana™miRNA mimic, Ambion, USA). To examine MiR-10b expression after transfection, total RNA was extracted from the transfected cells, and then cDNA was synthesized using TaqMan MicroRNA reverse transcription kit (ThermoFisher, USA) according the manufacturer’s instructions. The housekeeping gene GAPDH was used as the endogenous reference gene, and the PCR primer sequences [31] were 5′-ACCACAGTCCATGCCATCAC-3′ and 5′-TCCACCACCCTGTTGCTGTA-3′.

### Cell proliferation assay

Cell proliferation was analyzed by WST-1 assay (Roche, USA). Briefly, the transfected glioblastoma cells (including MiR-10b mimics, anti-MiR-10b inhibitor and random sequence MiRNA mimic) were seeded to 96-well plates at density of 2 × 10^4^ cells well^−1^ and cultured overnight. Then, different doses of ionizing radiation were used to treat the cells. Cells were continued to grow at 37 °C in a humidified incubator. Every 24 h of culture, 20 µl of WST-1 was added to each well and incubated for at least 60 min at 37 °C. Then, the absorbance was measured at 490 nm. All experiments were performed in triplicates.

### Caspase 3/7 activity

The glioblastoma cells were seeded in 24-well plates at density of 2 × 10^5^ cells well^−1^ and cultured overnight. Different doses of ionizing radiation were used to treat cells. After 24 h of culture, the caspase 3/7 activity was examined using the Caspase-Glo3/7 assay kit (Promega, Madison, USA) following the manufacturer’s protocol. Briefly, 20 µl of Caspase-Glo reagent was added to each well, then cells were incubated for at least 8 h with gentle shaking at room temperature. The luminescence value was measured using 1 min lag time and 0.5 s well^−1^ read time. All experiments were carried out in triplicates.

### TUNEL assay

Cell apoptosis were evaluated using Click-iT^®^ TUNEL Assay kit (Invitrogen, USA). Briefly, A172cells were seeded to 96-well plates at the density of 2 × 10^4^ cells well^−1^ and cultured overnight. Then, cells were treated with ionizing radiation (5 Gy). Following 24-h incubation, cells were fixed with 4 % paraformaldehyde in PBS at room temperature for 20 min. Then the cells were permeabilized with Triton X-100 for 20 min followed incubation with terminal deoxynucleotidyltransferase reaction buffer for at least 10 min at room temperature. TUNEL reaction mixture was added to each well and continued to incubate for 1 h at 37 °C. Following washes with 3 % BSA in PBS for 2 min with gentle shaking, Click-iT reaction mixture was added and incubated for 30 min at room temperature. Afterwards, the cell nuclei were counterstained using Hoechst 33342 for 15 min at room temperature. The positive nuclei were measured by counting the TUNEL-positive cells in eight different, random fields of view each well.

### Matrigel invasion assays

Cell migration and invasion was evaluated by Promega invasion assays as described before [32]. Briefly, transfected A172 cells were treated with 5 Gy of ionizing radiation, seeded on the upper transwell insert with or without matrigel in DMEM without FBS. DMEM containing 5 % FBS was added to the lower insert. After 18 h of culture, the invaded cells were stained by Diff-Quik stain after removal of the non-invaded cells with cotton swabs. The numbers of invaded cells were counted, and the percentage of migration and invasion was shown as a ratio of invaded cells over cells normalized on day 2 of growth curve.

### Western blot assay

The A172 cells were lysed in ice-cold lysis buffer (50 mM Tris–HCl, pH 7.5, 0.1 % SDS, 150 mM NaCl, 0.5 % deoxycholate, 1 % NP-40, and 1× protease inhibitors). Twenty microgram of protein lysates were loaded to the SDS-PAGE gel and transferred to PVDF membranes (Sigma, USA). Antibody incubations were carried out in 5 % non-fat dry milk in TBS-T buffer at 4 °C with primary antibodies [Bcl-2 (catalogue number: 2872, dilution: 1:1000), Bax (catalogue number: 2774, dilution: 1:1000), AKT (catalogue number: 9272, dilution: 1:1000) and p-AKT (catalogue number: 9271, dilution: 1:1000) from Cell Signaling Technology, USA]. The immune signals were developed with the EasySee Western Blot Kit (Transgen, Shanghai).

### Statistical analysis

Results were shown as mean ± standard deviation. SPSS program (version 11.0, IBM, USA) was used for statistical analyses using Student’s t test or ANOVA test. Differences are considered statistically significant if *p* < 0.05.
